# Comparison of Supervised Machine Learning Models to Logistic Regression Model Using Tooth-Related Factors to Predict the Outcome of Nonsurgical Periodontal Treatment

**DOI:** 10.3390/diagnostics15182333

**Published:** 2025-09-15

**Authors:** Ali J. B. Al-Sharqi, Mohammed Taha Ahmed Baban, Nada K. Imran, Sarhang S. Gul, Ali A. Abdulkareem

**Affiliations:** 1 Department of Periodontics, College of Dentistry, University of Baghdad, Bab Al Mudam, Baghdad P.O. Box 1417, Iraq; alijaaferalsharki@gmail.com (A.J.B.A.-S.); nada.k.omran@codental.uobaghdad.edu.iq (N.K.I.); ali.abbas@codental.uobaghdad.edu.iq (A.A.A.); 2 Department of Dental Nursing, Sulaimani Technical Institute, Sulaimani Polytechnic University, Sulaymaniyah P.O. Box 20-236, Iraq; mohammed.baban@spu.edu.iq; 3 Medical Laboratory Department, College of Health and Medical Technology, Sulaimani Polytechnic University, Sulaymaniyah P.O. Box 20-236, Iraq; 4 Department of Periodontics, College of Dentistry, University of Sulaimani, Sulaymaniyah 46001, Iraq

**Keywords:** periodontitis, nonsurgical periodontal treatment, tooth-related factors, machine learning models, logistic regression

## Abstract

**Background/Objectives:** Conventional logistic regression is widely used in the field of dentistry, specifically for prediction purposes in longitudinal studies. This study aimed to compare the validity of different supervised machine learning (ML) models to the conventional logistic regression (LR) model to predict the outcomes of nonsurgical periodontal treatment (NSPT). **Methods:** Patients diagnosed with periodontitis received full periodontal charting, including bleeding on probing (BoP), probing pocket depth (PPD), and clinical attachment loss (CAL). Furthermore, the tooth type, tooth location, tooth surface, arch type, and gingival phenotype were also collected as site-specific predictors. Later, root surface debridement was provided and treatment outcomes were evaluated after 3 months. Site-specific predictors were used to train five ML models, including random forest (RF), decision tree (DT), support vector classifier (SVC), K-nearest neighbors (KNN), and Gaussian naïve Bayes (GNB), to develop predictive models. **Results:** Site-specific predictors of 1108 examined sites were used, and the overall accuracy prediction of the conventional LR model was 70.4%, with PPD statistically significantly associated with the outcome of NSPT (odds ratio = 0.577, *p* = 0.001). Among the ML models examined, only GNB and SVC showed comparable prediction accuracy (71.0% and 70.4%, respectively) to the LR model, whereas the prediction accuracies of KNN, RF, and DT were 65.0%, 62.0%, and 61.0%, respectively. Similarly, baseline PPD was shown to be the most important featured predictor by both the RF and DT models. **Conclusions**: The evidence suggests that supervised ML models do not outperform the LR model in predicting the outcomes of NSPT. A larger sample size and more predictors of periodontitis are necessary to enhance the accuracy of ML models over the LR model in predicting the outcomes of NSPT.

## 1. Introduction

There is ample evidence supporting the efficacy of nonsurgical periodontal treatment (NSPT) for treating inflammatory periodontal disease, which is one of the most prevalent diseases affecting populations worldwide [1]. The term NSPT mainly refers to mechanical methods used to eradicate dysbiotic biofilms, calculus, and other debris coronally and from below the gingival line, performed with either manual or ultrasonic scalers [2]. NSPT is the recommended technique for treating periodontal pockets with shallow to moderate depth and reducing bacterial load from deep periodontal pockets at the early stage before surgical periodontal treatment [2]. Rapid improvement of clinical periodontal parameters, within the 12-week follow-up period after NSPT was observed, with marked reduction in the bacterial load of the subgingival and salivary microbiotas [3]. However, microbiological analysis of periodontal pockets 3 months after NSPT showed that the non-responding sites were dominated by the bacterial species *Porphyromonas gingivalis* and *Treponema denticola* [4]. The current guideline recommends another cycle of NSPT following step 1 and 2 therapy of residual pockets of 4–5 mm; however, for sites with residual pockets of ≥6 mm, surgical periodontal treatment is recommended [5].

It is acknowledged that the efficacy of NSPT is highly influenced by both patient- and tooth-related factors, with many variables identified [6,7,8,9,10,11]. The expected success rate could be more than 80% or less than 60% depending on a range of factors, including tooth type, anatomical landmarks, operator skill, genetics, smoking habits, uncontrolled diabetes, and pathogenicity of bacteria [1,12]. Indeed, identifying these factors in advance before providing NSPT is of paramount importance not only for the clinician evaluating the efficacy of NSPT but also in enabling them to provide patients with clearer information regarding expected outcomes, possible additional cycles of NSPT, or selecting other therapeutic options, such as surgical periodontal therapy in the future [2].

Researchers have examined several tooth-related factors as predictive indicators of NSPT, such as baseline probing pocket depth (PPD), clinical attachment loss (CAL), bleeding on probing (BoP), location of the tooth, number of roots, surface of the tooth, and arch [6]. However, evidence on the impact of tooth-related factors on the efficacy of NSPT is still inconclusive, and further studies are encouraged [13,14,15,16].

On the other hand, concern has been raised regarding periodontal data with an inherent hierarchical structure, which might pose difficulties for the statistical analysis. It has been reported that aggregation of site data for an individual patient and its expression as a mean might lead to masking of critical information and overestimation of the standard errors. In contrast, analyzing data at the tooth or site level without considering the interdependence of teeth or sites within the same patient can lead to an underestimation of the standard error. One way to address this issue is by using multilevel modeling—a statistical technique originally developed in educational research, but now widely applied across various areas of health sciences [17,18].

The implication of logistic regression (LR) is that it allows the impact of different tooth-related factors on the outcome of NSPT to be identified. Furthermore, the use of a backward stepwise method in LR helps to exclude redundant parameters without affecting the overall accuracy of the model. Thus, this conventional statistical test is widely used in the field of dentistry, specifically for prediction purposes in longitudinal studies [19,20]. Additionally, predictive models frequently lack externally validation, leaving uncertainty about whether these algorithms can be reliably applied to other diverse populations.

Recently, machine learning (ML) models have gained growing attention, as they rely on developing algorithm and statistical models from data rather than direct programming for statistical analysis. ML models offer significant benefits for analyzing large-scale, complex, and diverse data commonly produced in biomedical research. While their use in dental data analysis is still emerging, they show strong potential for improving the accuracy of diagnostic pattern recognition [21,22]. Furthermore, recent systematic reviews that have examined studies using ML models for periodontal screening, diagnosis, and prognosis found that supervised ML has potential for use in prognostic models applied in periodontology; however, the current evidence is not conclusive [23,24].

Supervised ML models, such as decision tree (DT), K-nearest neighbors (KNN), random forest (RF), support vector classification (SVC), and Gaussian naïve Bayes (GNB) have been used for diagnostic and prognostic purposes based on the input data [24,25,26]; however, the reliability and accuracy of these models in comparison to the most widely used LR statistical test have yet to be examined [27]. Therefore, the current study aimed to compare the accuracy of the above supervised ML models to the LR model using the baseline data of several tooth-related factors as a “typical” dataset to predict the outcome of NSPT. It is important to acknowledge that the tooth-related factors used in this study were solely included for the purpose of comparison between supervised ML models and LR rather than actual prediction of NSPT.

## 2. Materials and Methods

### 2.1. Study Design

This cohort study included patients with periodontitis seeking periodontal treatment who were recruited consecutively by the Department of Periodontics, College of Dentistry, University of Baghdad between April 2024 and April 2025. Ethical rules declared by the World Medical Association Declaration of Helsinki and the later amendments were followed in the current study. Initially, ethical approval for conducting this study was issued by the Ethics Committee, College of Dentistry, University of Baghdad (Reference No. 663). The purpose of the study, details of the intended treatment plan, and other relevant information were fully declared to each patient before signing a consent form.

### 2.2. Eligibility Criteria

The inclusion criteria were patients aged ≥18 years with no history of systemic diseases, such as diabetes mellitus, that would affect NSPT. The recruited patients needed to be diagnosed with periodontitis, regardless of the stage and grade, with 10 pairs of occluding teeth. The case definition of periodontitis as stated by the American Academy of Periodontology/European Federation of Periodontology was followed in this study. Evidence of interproximal CAL in ≥2 non-adjacent teeth, or presence of a detectable CAL ≥ 3 mm combined with PPD ≥ 4 mm on the buccal/lingual surfaces were the criteria used to diagnose periodontitis [28]. Periodontal pockets included in this study needed to exhibit a PPD between 4 and 6 mm, with no furcation involvement, as indications for NSPT. Current smokers, pregnant women, those receiving recent or ongoing periodontal treatment, and patients not willing to consent were excluded from this cohort.

### 2.3. Tooth-Related Parameters and Intervention

Periodontal clinical parameters, including BoP [29], PPD, CAL, and gingival phenotype, were recorded for each patient at baseline and after 3 months by the same clinician (A.J.B.A.) at four surfaces per tooth, using the UNC-15 periodontal probe (Medesy, Maniago, Italy). Absence or presence of BoP was recorded as 0 or 1, respectively. Simultaneously, PPD and CAL were measured, representing the linear distances from the gingival margin and cementoenamel junction, respectively, to the base of the sulcus/pocket.

Gingival phenotype was determined using the Colorvue™ Biotype Probe (Hu-Friedy^®^, Chicago, IL, USA), which is a set of three probes with different color coding, used in a sequential order to define the phenotype [30]. In detail, the white-colored probe was first inserted in the gingival crevice, and if the color was visible through the gingiva, the phenotype was thin. If the transparency of the white color through the gingiva was completely blocked, the same procedure was repeated with the green-colored probe, which indicated medium phenotype if the color was apparent. If not apparent, the blue tip probe was inserted in a similar manner. Transparency of the blue color through the gingiva indicated a thick phenotype, whereas if the blue color was not visible, the gingival tissue was classified as very thick. These last two thickness possibilities were considered as a thick phenotype group. Furthermore, the tooth-related factors collected were tooth type (single-rooted or multirooted teeth), tooth location (incisors, premolars, and molars), arch type (maxillary and mandibular teeth), and tooth surfaces (mesial, distal, facial, and oral surfaces).

Oral hygiene instructions were provided for each patient, followed first by professional mechanical plaque removal using an ultrasonic scaler (Woodpecker, Ultrasonic Piezoelectric Scaler UDS-A, Guilin, China) and then by polishing the teeth. The patients were instructed to return after one week to receive root surface debridement, using Gracey curettes (Medesy, Maniago, Italy) for all periodontal pockets. Similar types of tooth brush, toothpaste, and interdental brush (Curaprox, Kriens, Switzerland) were provided for each patient. All measurements were performed by the same calibrated examiner, using a UNC-15 periodontal probe (Medesy, Maniago, Italy). The level of consistency for categorical parameters (BoP) was >80% using the kappa coefficient test, and for continuous parameters (PPD and CAL), it was >90%, as indicated by the interclass coefficient test.

### 2.4. Outcomes and Sample Size

Reduction of the PPD to ≤4 mm without BoP [31] after 3 months was set as the primary outcome indicating the success of NSPT. Secondary outcomes included BoP, CAL, gingival phenotype, tooth type, tooth location, arch type, and tooth surfaces. Accordingly, successful/unsuccessful pocket closure was accounted for when calculating the priori sample size. Data from a previous study indicated a successful/unsuccessful ratio of NSPT as 1.6 [6], with 0.05 α-error, and 0.8 power. The estimated sample size was 65 patients, which was rounded to 72 patients to allow for 10% dropout.

### 2.5. Statistical Analysis

#### 2.5.1. Conventional Statistical Analysis

Mean, standard deviation, frequency, and percentage were used to describe continuous and categorical variables. The Shapiro–Wilk test was used to determine whether the data were parametric or non-parametric. Differences in the PPD and CAL clinical parameters between the baseline and endpoint were determined using paired *t*-tests, while a chi-squared test was performed to detect differences between categorical clinical parameters of BoP, gingival phenotype, tooth type, tooth location, arch type, and tooth surfaces. A binary LR model was used to determine the potential of the tooth-related clinical parameters to predict the outcomes of NSPT. The dependent variable was the successful/unsuccessful NSPT, which was dichotomized into 1 or 0, respectively. For the binary or categorical tooth-related factors (BoP, gingival phenotype, tooth type, tooth location, arch type, and tooth surfaces), the first variables were used as a reference. The level of significance was set at a *p* value less than 0.05, and all statistical analyses were conducted using GraphPad Prism (GraphPad Prism Software, version 9, San Diego, CA, USA) and SPSS (version 26, IBM, Boston, MA, USA) software.

#### 2.5.2. Machine Learning Models Algorithms

All ML models in this study were developed and evaluated on a Lenovo laptop (Model: 20M3, Lenovo group limited, Beijing, China) equipped with an 8th-generation Intel Core i5 processor (Intel corporation, Chandler, AZ, USA). Five ML models of DT, RF, KNN, SVC, and GNB were applied. The dataset was randomly divided into training (80%) and testing (20%) subsets. Feature importance analysis was conducted to identify the most influential predictors underlying model decisions. Model performance was assessed using confusion matrices and ROC curves for each model. To ensure robustness, tenfold cross-validation with random shuffling and ten repetitions was employed, and the mean accuracy and standard deviation were calculated. All procedures, including data preprocessing, model training, evaluation, and visualization, were conducted using Python software, version 3.13.5, developed by the Python Software Foundation, Wilmington, DE, USA.

## 3. Results

### 3.1. Basic Demographic and Tooth-Related Parameters

**Figure 1:**
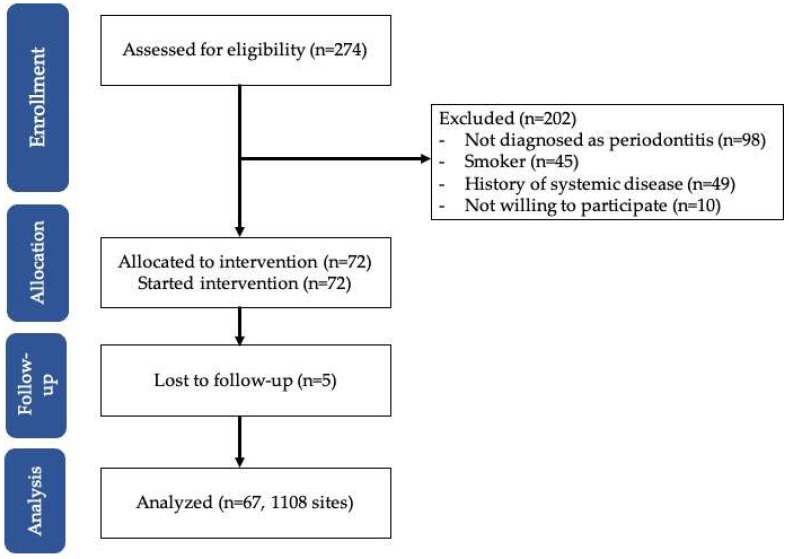
Flow diagram of the study.

After applying the eligibility criteria, 72 periodontitis patients were recruited (out of 274). A total of 5 patients did not commit to the planned appointment, leaving 67 patients for the final analysis (Figure 1).

The mean age of recruited patients (33 male and 34 female) was 56.72 ± 7.10 years old (Table 1). The clinical periodontal parameters PPD, CAL, and BoP were significantly improved 3 months after NSPT. Out of 1108 sites examined, 781 (70.49%) periodontal pockets exhibited pocket closure as a successful outcome (Table 1). On the other hand, when the baseline tooth-related factors were analyzed versus their outcome (successful or unsuccessful) after 3 months, only baseline PPD and tooth type (single-rooted vs. multirooted teeth) showed statistically significant differences (Table 2).

### 3.2. Logistic Regression Model

Ability of tooth-related factors to predict the outcomes of NSPT was assessed by a binary LR model (Table 3). The results indicate that only PPD was statistically significantly associated with outcomes of NSPT (odds ratio = 0.577), with a negative estimated coefficient. This suggests that increasing the PPD at baseline was a predictor of unsuccessful treatment outcome after 3 months and vice versa. No other clinical tooth-related factors showed any significant effect on the outcomes of NSPT. Lastly, the overall accuracy of this model was 70.4%.

### 3.3. Machine Learning Models

**Figure 2:**
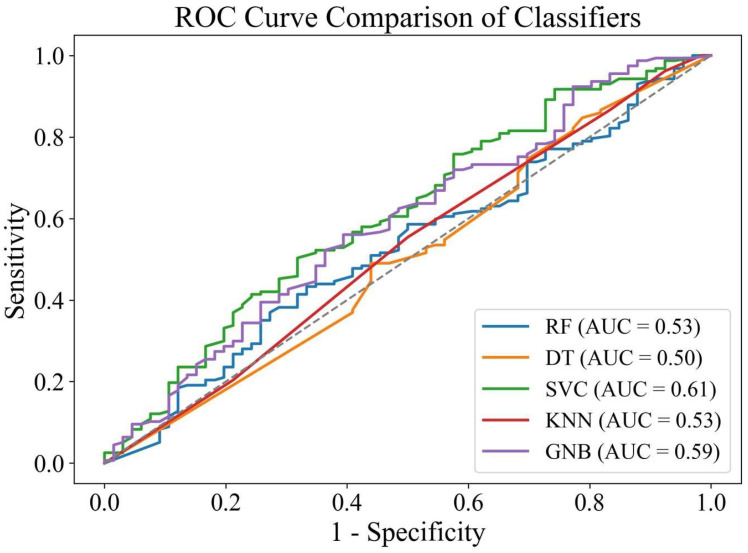
ROC and AUC of tested ML models to predict the outcome of NSPT after 3 months (the reference line is presented as dashed line).

Amongst the ML models, SVC and GNB showed the highest AUCs of 0.61 and 0.59, respectively, indicating their ability to differentiate the successful and unsuccessful treatment outcomes of NSPT after 3 months. In contrast, both the RF and KNN models demonstrated an AUC of 0.53, and the lowest AUC (0.5) was shown by DT (Figure 2).

**Figure 3:**
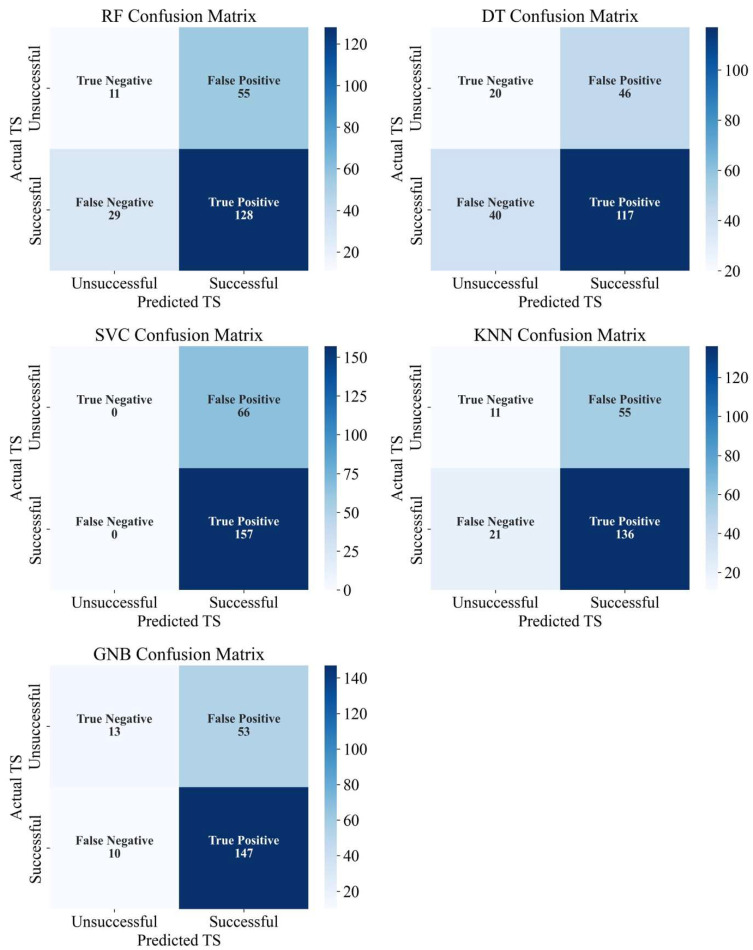
Confusion matrices of examined ML models to predict the outcomes of NSPT.

It is apparent that GNB and SVC had the highest testing accuracies of 0.71 and 0.704, respectively, with the lowest training accuracy (GNB = 0.72, SVC = 0.703) (Table 4). Furthermore, the confusion matrix of GNB was able to correctly identify 13 out of 66 unsuccessful sites and 147 out of 157 successful sites. In contrast, the confusion matrix of SVC did not identify any unsuccessful sites and was able to identify all successful sites (Figure 3). RF and DT had the highest training accuracies (0.81 both) with the lowest testing accuracies of 0.62 and 0.61, respectively. The confusion matrix of RF only identified 11 out of 66 unsuccessful sites and 128 out of 167 successful sites, while the DT confusion matrix was able to identify 20 out of 66 unsuccessful sites and 117 out of 167 successful sites. Although SVC and GNB recorded the highest f1-scores of 0.83 and 0.82 for successful sites, the f1-scores of SVC and GNB for the unsuccessful sites were 0 and 0.29, respectively. Among the ML models, DT had the highest f1-score for unsuccessful sites (0.32). The precision, recall, and f1-scores, as well as the training accuracy and the testing accuracy of all the ML models are presented in Table 4. Additionally, the results for the confusion matrices of tested ML models are presented in Figure 3.

**Figure 4:**
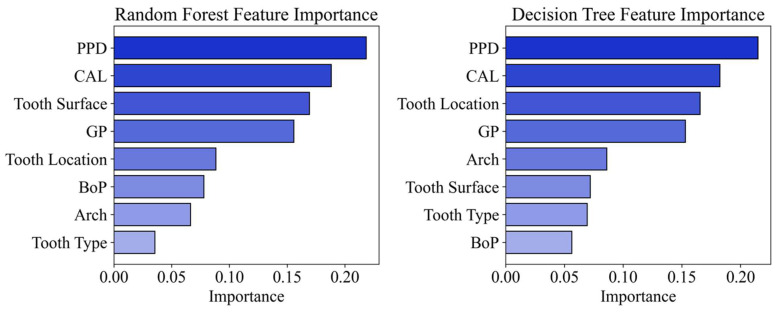
The most important predictors of NSPT as shown by RF and DT models. PPD: probing pocket depth, CAL: clinical attachment loss, GP: gingival phenotype, BoP: bleeding on probing.

On the other hand, examination by RF and DT of the importance of tooth-related factors in contributing to the prediction of the outcome of NSPT showed that PPD and CAL are the most important predictors for both models. Next, tooth surfaces and tooth location were identified as the third most important tooth-related factors by the RF and DT models, respectively. Finally, gingival phenotype was shown to be the fourth most important tooth-related factor by both models (Figure 4).

Tenfold cross-validation was used to examine the performance of all tested ML models. Similarly, GNB (0.713 ± 0.037) and SVC (0.7 ± 0.011) demonstrated the highest accuracy, followed by RF (0.667 ± 0.049) and KNN (0.662 ± 0.032), whilst DT showed the lowest accuracy (0.613 ± 0.057) (Table 5).

### 3.4. Logistic Regression vs. Machine Learning Models

Comparison between the LR model and ML models can be performed via assessing their overall accuracy and the most important tooth-related factors contributing to their prediction capabilities. It is apparent that the overall accuracy of LR (70.4%, Table 3) was almost equal to that recorded by GNB (71%) and SVC (70.4%), as shown in Table 4 and again following tenfold cross-validation (Table 5). Moreover, in the case of the LR model, among the tooth-related factors, only PPD had a statistically significant association with the outcome of NSPT (Table 3), which is similar to the corresponding results for the RF and DT models (Figure 4).

## 4. Discussion

Supervised ML models have recently been used for predictive purposes in medicine [32]. The data concerning the use of supervised ML models for prediction purposes in dentistry are inconclusive, as no comparative data were used to evaluate the efficacy of supervised ML models against the traditional risk assessment tools and LR model [24,27]. Consequently, this study was carried out to compare the efficacy of the recent supervised ML models to the most commonly used statistical tests, namely LR with a typical dataset of tooth-related factors. The results of the current study show that the GNB and SVC models have prediction accuracy comparable to that of the LR model regarding outcomes of NSPT. Additionally, similarly to LR, both the RF and DT models identified PPD as the most important factor for prediction of the outcomes of NSPT.

Indeed, a huge number of studies have investigated various biomarkers, such as microbials, host-related markers in saliva, and gingival crevicular fluid for prediction of the outcomes of NSPT [33,34,35]. Moreover, as treatment outcomes may not only differ between patients but also between different sites of the same tooth within the same patient [36,37,38], researchers have examined the most convenient clinical and tooth-related factors that might also have an impact on the outcomes of NSPT [6,12,13].

Introducing different clinical, molecular, host-derived, and microbiological biomarkers into dentistry for prediction, diagnosis, and monitoring dental and periodontal/peri-implant diseases has led to increasing interest [39,40]. Among clinical parameters, the severity of calculus accumulation and suppuration were suggested as predictors of tooth survival [41]. Baseline shallow pockets with interproximal location were suggested as predictors of NSPT success [42]. The latter result was consistent with findings of this study indicating shallow baseline PPD has an impact on the outcomes of NSPT. Indeed, periodontal pockets with shallow or moderate depth (<6 mm) are easier to access and debride than deeper pockets, which are inaccessible to mechanical cleaning and highly infected with pathogenic Gram-negative bacteria [43]. The key factors for success of periodontal therapy are efficient debridement of subgingival dysbiotic microbiota, severity of disease, absence of local/systemic risk factors, compliance to recall visits, and having proper oral hygiene measures [12,44].

The definitions of successful and non-successful NSPT vary according to its application at the patient level and site-specific level. For example, at the patient level, for the baseline PPD of 4–6 mm, a mean PPD reduction of around 1.29 mm was expected [45], whereas, for the site-specific level, as used in the current study, a reduction of PPD to ≤4 mm without BoP after 3 months was set as the primary outcome indicating the success of NSPT [31].

This study used the most common tooth-related factors of PPD, CAL, BoP, gingival phenotype, tooth type, arch, tooth location, and tooth surfaces to predict the outcomes of NSPT by the LR model and supervised ML models. Both the conventional LR and ML models demonstrated moderate prediction abilities (around 70%), and with such limited prediction abilities, the clinical implications of the current study might be arguable. However, it is worth mentioning that the purpose of this study was merely to compare the conventional LR statistical test to ML models rather than assessing the actual prediction abilities of the above tooth-related factors regarding NSPT outcomes.

It is important to acknowledge that both the DT and RF models indicated PPD as the most important prediction parameter of outcomes of NSPT, which aligns with the finding for the LR model that only PPD had a statistically significant association with NSPT outcomes. Interestingly, CAL was shown to be the second most important tooth-related factor by both the DT and RF models. However, the LR model did not demonstrate a statistically significant association of CAL with NSPT outcomes. This can be explained by the strong association between PPD and CAL as clinical parameters and, as the prediction role of CAL might be already explained by PPD, CAL would have no significant impact on the overall prediction rate of LR. Similarly, the responses of different gingival phenotypes to biofilm-induced periodontal disease ranged from CAL in the thin, scalloped phenotype to the formation of deep PPD in the thick, flat phenotype [46]. Again, the non-statistically significant association of gingival phenotype to the outcomes of NSPT may already have been explained by PPD.

Indeed, choosing parameters with strong mutual associations hinders the overall prediction ability of LR and, therefore, selecting such parameters for prediction purposes when applying the LR model is highly discouraged. Additionally, it has been reported that the LR model can lead to oversimplification, especially in complex diseases, such as periodontitis, which are characterized by intricate interactions between variables and outcomes [32,47,48]. In contrast, in ML models (RF and DT), such overfitting of the model may not strongly affect the overall prediction result, and consequently, such parameters (CAL and gingival phenotype) are still presented among the most important parameters affecting the prediction accuracy of ML models. On the other hand, the outcomes of NSPT after 3 months have been shown to differ statistically according to the tooth type (single-rooted vs. multirooted teeth). The possible explanation could be that the single-rooted teeth can be cleaned easily due to the absence of possible furcation involvement, which causes difficulties during NSPT [49]. However, the LR model did not show a statistically significant association of tooth type with NSPT outcomes; again, both the DT and RF models ranked tooth type as the least important parameter in predicting the outcomes of NSPT. A possible explanation could be that many algorithms with different mechanisms are used to train DT and RF models rather than a static algorithm (paired *t* test) to compare between successful and unsuccessful sites [50].

These results indicate that ML models have similar or lower capabilities for NSPT prediction 3 months after treatment, based on the above-mentioned tooth-related factors. These results are not commensurate with the results of the current systematic reviews showing that supervised ML methods can demonstrate better performance than traditional statistical tests for periodontal screening, diagnosis, disease progression, and prognosis [23,24]. This can be explained by the fact that the primary outcome of the current study is that NSPT is fundamentally different from periodontal screening and diagnosis. Thus, caution should be used when comparing the same ML models in applications of different prediction models, as well as when different independent parameters are used.

Although internal validation of the models was performed, and GNB and SVC produced comparable results to the LR, the lack of external validation and using data of one population are considered as the main limitations of the current study and hence, generalization of the results should be avoided. In fact, this highlights the need for external validation of the ML models in other heterogeneous populations, using larger datasets to confirm their efficacy in a real-world scenario. Nonetheless, this is the first study to compare the efficacy of ML models to conventional LR in predicting the outcomes of NSPT using tooth-related factors, and it could pave the way for future studies to apply ML models with various predictors, such as patient-related, microbial, and host-related factors, to enhance the overall predictive accuracy of ML models.

## 5. Conclusions

Available evidence indicates that, in predicting outcomes of NSPT, supervised ML models do not offer substantial advantages over the conventional LR model. Therefore, considering its transparency and interpretability, LR remains the suitable and often preferable modeling approach in clinical settings, particularly in the absence of large, high-dimensional datasets. Certainly, to effectively leverage the capabilities of ML models in predicting the outcomes of NSPT, it is imperative to employ larger sample sizes alongside more complex predictors. Moreover, any predictive models developed should be subjected to rigorous external validation prior to making assertions regarding their generalizability or potential impact on broader populations.

## Figures and Tables

**Table 1:** Basic demographic and clinical characteristics of the study population (*n* = 67).

Variables	Baseline	Endpoint
Age † (years)	56.72 ± 7.10	
Sex ‡		
Male	33, 49.30%	
Female	34, 50.70%	
PPD † (mm)	4.74 ± 0.66	3.83 ± 0.69 *
CAL † (mm)	3.57 ± 0.66	3.19 ± 0.61 *
BoP ‡		
No bleeding	231, 20.85%	880, 79.42% **
Bleeding	877, 79.15%	228, 20.58% **
Gingival phenotype ‡		
Thin	142, 12.82%	
Medium	642, 57.94%	
Thick	324, 29.24%	
Tooth type ‡		
Single-rooted	696, 62.82%	
Multirooted	412, 37.18%	
Tooth location ‡		
Incisors	413, 37.27%	
Premolars	371, 33.49%	
Molars	324, 29.24%	
Arch ‡		
Maxillary	637, 57.49%	
Mandibular	471, 42.51%	
Tooth surface ‡		
Mesial	497, 44.86%	
Distal	487, 43.95%	
Facial	116, 10.47%	
Oral	8, 0.72%	
Site-specific outcomes (*n* = 1108) ‡		
Successful		781, 70.49%
Unsuccessful		327, 29.51%

BoP: bleeding on probing, CAL: clinical attachment level, PPD: probing pocket depth. † Mean ± SD; ‡ frequency, percent; * significant difference at *p* < 0.05 using paired *t*-test; ** significant difference at *p* < 0.05 using paired chi-squared test.

**Table 2:** Comparison of baseline tooth-related factors in successful (*n* = 782) and unsuccessful (*n* = 329) sites.

Tooth-Related Factors	Successful	Unsuccessful	*p* Value
PPD † (mm)	4.65 ± 0.61	4.96 ± 0.71	<0.001 *
CAL † (mm)	3.57 ± 0.66	3.57 ± 0.64	0.9
BoP ‡			
No bleeding	621, 56.05%	256, 23.10%	0.57
Bleeding	159, 14.35%	72, 6.50%	
Gingival phenotype ‡			
Thin	100, 9.03%	42, 3.79%	0.93
Medium	450, 40.61%	192, 17.33%	
Thick	231, 20.85%	93, 8.39%	
Tooth type ‡			
Single-rooted	510, 46.03%	186, 16.79%	0.01 **
Multirooted	271, 24.46%	141, 12.72%	
Tooth location ‡			
Incisors	309, 27.80%	105, 9.48%	0.054
Premolars	255, 22.82%	118, 10.65%	
Molars	218, 19.68%	106, 9.57%	
Arch ‡			
Maxillary	436, 39.35%	201, 18.14%	0.11
Mandibular	344, 31.05%	127, 11.46%	
Tooth surface ‡			
Mesial	332, 29.96%	165, 14.89%	0.10
Distal	356, 32.13%	131, 11.83%	
Facial	87, 7.85%	29, 2.62%	
Oral	5, 0.45%	3, 0.27%	

† Mean ± SD; ‡ frequency, percent; * significant difference at *p* < 0.05 using paired *t*-test; ** significant difference at *p* < 0.05 by using chi-squared test.

**Table 3:** Logistic regression for predictors of site-specific outcome for nonsurgical periodontal treatment.

Predictors	B	SE	*p* Value	Exp (B)	95% CI for EXP (B)
PPD	−0.550	0.103	0.001	0.577	0.471 to 0.706
CAL	0.067	0.102	0.51	1.070	0.875 to 1.307
BoP ^a^					
Bleeding	−0.28	0.169	0.098	0.75	0.542 to 1.053
Gingival phenotype ^b^					
Medium	0.009	0.234	0.971	1.009	0.637 to 1.579
Thick	0.049	0.167	0.772	1.05	0.756 to 1.457
Tooth type ^c^					
Multirooted	0.4	0.281	0.155	1.491	0.86 to 2.585
Tooth location ^d^					
Premolars	−0.348	0.346	0.314	0.706	0.358 to 1.391
Molars	−0.399	0.277	0.149	0.671	0.39 to 1.154
Arch ^e^					
Mandibular	−0.118	0.153	0.44	0.889	0.659 to 1.199
Tooth surface ^f^					
Distal	−0.16	0.741	0.983	0.984	0.23 to 4.206
Facial	0.261	0.743	0.725	1.299	0.303 to 5.568
Oral	0.28	0.767	0.715	1.323	0.294 to 5.942
Accuracy of the model	70.4%				

B: estimated coefficient, SE: standard error, Exp (B): odds ratio, CI: confidence interval, PPD: probing pocket depth, CAL: clinical attachment loss. Reference: (^a^) non-bleeding, (^b^) thin, (^c^) single-rooted, (^d^) incisors, (^e^) maxillary, (^f^) mesial.

**Table 4:** The precision, recall, f1-score, training accuracy, and testing accuracy of the ML models to predict the outcomes of NSPT.

ML Models	Treatment Outcome	Precision	Recall	F1-Score	Training Accuracy	Testing Accuracy
Random forest	Unsuccessful	0.28	0.17	0.21	0.81	0.62
	Successful	0.7	0.82	0.75		
Decision tree	Unsuccessful	0.33	0.3	0.32	0.81	0.61
	Successful	0.72	0.75	0.73		
Support vector classifier	Unsuccessful	0	0	0	0.703	0.704
	Successful	0.7	1	0.83		
K-nearest neighbors	Unsuccessful	0.34	0.17	0.22	0.76	0.65
	Successful	0.71	0.87	0.78		
Gaussian naïve Bayes	Unsuccessful	0.57	0.2	0.29	0.72	0.71
	Successful	0.73	0.94	0.82		

**Table 5:** Tenfold cross-validation for the testing accuracy of all the ML models.

Training Set	RF	DT	SCV	KNN	GNB
1	0.642	0.625	0.705	0.651	0.687
2	0.693	0.630	0.702	0.693	0.684
3	0.657	0.621	0.702	0.594	0.720
4	0.639	0.603	0.666	0.657	0.648
5	0.621	0.522	0.702	0.657	0.720
6	0.621	0.576	0.702	0.666	0.720
7	0.621	0.540	0.702	0.639	0.720
8	0.702	0.603	0.702	0.648	0.729
9	0.747	0.675	0.702	0.693	0.792
10	0.747	0.729	0.711	0.720	0.747
Mean ± SD	0.667 ± 0.049	0.613 ± 0.057	0.7 ± 0.011	0.662 ± 0.032	0.713 ± 0.037

## Data Availability

The data that support the findings of this study are available from the corresponding author upon reasonable request due to ethical reasons.
